# Application value of triglyceride-glucose index and triglyceride-glucose body mass index in evaluating the degree of hepatic steatosis in non-alcoholic fatty liver disease

**DOI:** 10.1186/s12944-023-01954-5

**Published:** 2023-11-03

**Authors:** Mengyuan Wang, Mingxing Chang, Peipu Shen, Wei Wei, Huayao Li, Guifang Shen

**Affiliations:** Department of Health Management Center, The Affiliated Hospital of Xuzhou Medical University, Xuzhou, Jiangsu China

**Keywords:** Non-alcoholic fatty liver disease, TyG, TyG-BMI, Hepatic steatosis

## Abstract

**Background:**

The elevation of TyG is considered an important factor in promoting the progression of non-alcoholic fatty liver disease (NAFLD), but its impact on the degree of liver steatosis remains unclear. This study aims to explore the relationship between TyG and TyG-related indices, such as triglyceride glucose-body mass index (TyG-BMI), with the degree of liver fat accumulation.

**Methods:**

From January 2021 to March 2022, 1171 participants underwent health check-ups, and all underwent FibroScan transient elastography. The analysis focused on identifying the factors that contribute to the onset of NAFLD and the degree of hepatic steatosis.

**Results:**

The predictive value of TyG-BMI (OR = 1.039, 95% CI 1.031–1.046) in triggering NAFLD development was greater than that of TyG alone. The areas under the curve for TyG-BMI and TyG were calculated at 0.808 and 0.720, respectively. TyG-BMI (OR = 1.034, *P* < 0.001) was identified as a main independent factor affecting hepatic steatosis severity. With each incremental increase in TyG-BMI, the likelihood of experiencing an increase in the extent of hepatic steatosis was 1.034 times higher than that of the preceding unit.

**Conclusions:**

The TyG-BMI showed higher accuracy in predicting NAFLD than did the TyG, and was more closely linked to the severity of hepatic steatosis. Therefore, it can be included as a parameter in health management centers and should be widely used to screen and evaluate patients with NAFLD.

**Supplementary Information:**

The online version contains supplementary material available at 10.1186/s12944-023-01954-5.

## Background

Non-alcoholic fatty liver disease (NAFLD) is a chronic condition that is becoming more prevalent globally, with approximately 25% of adults worldwide being affected [1, 2]. The initial manifestation of this disease entails the excessive accumulation of lipids within hepatocytes, potentially advancing to non-alcoholic steatohepatitis. In severe cases, NAFLD may progress to cirrhosis or hepatocellular carcinoma [3]. Consequently, early recognition of risk factors for NAFLD and detection of fatty liver severity are of utmost importance.

The progression of NAFLD is the outcome of a blend of genetic and ecological elements. The most potent genetic risk factors for fatty liver disease are missense variations in PNPLA3 and TM6SF2. Both of these variations are connected with the full range of NAFLD [4]. Studies have substantiated the correlation between the TM6SF2 E167K variation and the content of fat in the liver, as well as the severity of liver disease [5]. Moreover, there exists a close association between hepatic steatosis and insulin resistance (IR) [6]. The key feature of NAFLD manifests in the excessive accumulation of triglycerides (TG) inside the liver, which in turn induces IR. Furthermore, the presence of IR gives rise to an excess secretion of fasting plasma glucose (FPG) and very low-density lipoprotein (VLDL) [7]. The metabolic pathways of TG in hepatocytes include de novo lipogenesis (DNL), oxidative breakdown, absorption, and transport. Any abnormalities in these pathways can lead to lipid deposition [8]. Excessive accumulation of highly toxic free fatty acids (FFAs) linked to IR leads to the mobilization of a large quantity of FFAs from adipose tissue and an increase in hepatic FFA synthesis for glucose synthesis, representing “the first step” in NAFLD [9]. Currently, classic indicators of IR, like the high insulin-normal glucose clamp test and homeostasis model assessment (HOMA) of IR, have limitations in their use owing to insulin instability and relatively high costs. Therefore, some primary hospitals and health management centers have not yet implemented them, making them difficult to use widely in clinical practice [10]. Additionally, they are not suitable for routine inspections and large-scale epidemiological investigations. Ascertaining individuals afflicted with NAFLD during its early stages is currently a focal point in research, hence necessitating the exploration of a straightforward and efficacious approach.

Recently, some scholars have found that a simple index, the triglyceride-glucose index (TyG), formed by taking the logarithm of the multiplication of TG and glucose, can effectively reflect IR and even outperform HOMA as a predictor for NAFLD [11]. Elevated TyG levels correlate with an augmented likelihood of NAFLD advancement and a reduced probability of NAFLD amelioration [10]. Moreover, researchers have noted that the amalgamation of body mass index (BMI) with the TyG demonstrates a more accurate assessment of IR in comparison to solely utilizing the TyG [12]. Combining the TyG with anthropometric measurements of obesity can predict liver fat deposition more comprehensively than using the TyG alone [13].

Transient elastography (TE) of the liver is a non-invasive means for evaluating the severity of fat deposition by evaluating controlled attenuation parameters (CAP) [14]. CAP can detect > 5% liver fat deposition and accurately distinguish between mild and moderate-to-severe liver fat deposition [15]. While research has been conducted regarding the correlation between TyG and NAFLD, there is limited research on the capacity of TyG or TyG-related parameters to determine the extent of liver fat accumulation. This study utilized FibroScan transient elastography to evaluate hepatic steatosis levels and examine the potential utility of TyG and TyG-BMI in predicting its severity. Additionally, the study explored whether TyG-BMI outperforms TyG in forecasting hepatic steatosis severity. The results of this investigation offer fresh perspectives for the management of NAFLD.

## Materials and methods

### Data source

Participants were selected among individuals who underwent health check-ups at the Health Management Center of Xuzhou Medical University Affiliated Hospital between January 1, 2021, and March 31, 2022. The inclusion criterion was the participants’ data, including TE and metabolic-related indices, being true and complete. The following populations were not included: (1) age < 18 years; (2) history of excessive alcohol consumption, equivalent to > 20 g/day for males or 10 g/day for females; (3) any history of liver diseases (such as hepatitis B, C) and other specific diseases that may result in fatty liver; (4) NAFLD combined with malignant tumors; (5) pregnancy or lactation; (6) recent use of drugs that affect liver or kidney metabolism.

The classification of NAFLD based on the CAP value of FibroScan was determined as follows: CAP value of ≥ 238 dB/m defined hepatic steatosis, CAP values between 238 and < 259 dB/m defined mild hepatic steatosis, those between 259 and < 292 dB/m defined moderate hepatic steatosis, those ≥ 292 dB/m defined severe hepatic steatosis.

The study included 1,171 individuals who underwent health examinations (943 males and 228 females) after the screening process. The ethics committee of the aforementioned hospital (XYFY2023-KL245-02) granted approval for this study.

## Methods

### General information and biochemical indicators

General information about the enrolled patients was collected. Height and weight were measured using a Shen Yuan HGM-601 measuring instrument. A pulse-wave blood pressure monitoring device was employed to measure blood pressure. After fasting blood collection, the levels of alanine aminotransferase (ALT), aspartate aminotransferase (AST), creatinine (CREA), uric acid (UA), total cholesterol (TC), TG, high-density lipoprotein (HDL-C), low-density lipoprotein (LDL-C), and FPG were determined using the Beckman AU5800 automatic biochemical analyzer. TyG = Ln[TG(mg/dL)* FPG (mg/dL)/2],TyG -BMI = TyG *BMI.

### TE

FibroScan PRO (produced by Shenzhen Hui Bo Medical Device Co., Ltd.) was used to assess the extent of liver steatosis by measuring the CAP values. Prior to the examination, participants were required to fast for at least 2 h and lie in a supine position whereby their right upper limb was extended and positioned towards the posterior aspect of their cranium. The investigator subsequently positioned the probe in a vertical manner on the surface of the skin within the intercostal region. The examiner triggered the device to begin collecting images and measurements by pressing a button on the probe. The final outcome was the median value obtained from 10 successful measurements. Notably, it is required that the success rate of the operation is no less than 60%, and the interquartile range (IQR) divided by the median (M) of the liver stiffness measurement (LSM) is ≤ 0.3. However, even if IQR/M is > 0.3, the result is considered relatively reliable when LSM is < 7.1 kPa [16].

### Statistical analysis

The descriptive statistics for normally distributed continuous data were reported as mean ± standard deviation. Skewed continuous data were presented the statistics as M (1/4, 3/4). To compare normally distributed continuous data and non-normally distributed continuous data, independent t-tests and Mann–Whitney U tests were utilized, respectively. Binary logistic regression models were utilized for the purpose of identifying the factors associated with NAFLD, while ordinal logistic regression models were employed to ascertain the factors associated with the severity of liver steatosis. The predictive abilities of TyG and TyG-BMI for NAFLD were estimated using the area under the receiver operating characteristic curve (AUROC), and the AUROC comparison was conducted using MedCalc. Correlation analysis between CAP and TyG, TyG-BMI, and their components was performed by GraphPad. The intergroup differences between CAP and the quartiles of TyG and TyG-BMI, as well as between TyG, TyG-BMI, and different degrees of fatty liver, were analyzed by Tukey’s multiple comparisons test. The criterion for statistical significance was defined as a *P*-value less than 0.05.

## Results

### Baseline characteristics of the study population

This study enrolled 1,171 participants, including 943 males and 228 females. Among them, 434 participants did not have NAFLD and 737 had NAFLD. Compared to the non-NAFLD group, patients with NAFLD, regardless of sex, demonstrated significantly elevated levels of systolic blood pressure (SBP), diastolic blood pressure (DBP), BMI, AST, ALT, TG, TC, UA, Cr, LDL, FPG, TyG, and TyG-BMI; in contrast, the non-NAFLD group exhibited a significantly higher level of HDL compared to the NAFLD group (Table 1).

**Table 1:** Baseline characteristics of subjects

	Male			Female		
	Non-NAFLD *n* = 306	NAFLD *n* = 637	*P* value	Non-NAFLD *n* = 128	NAFLD *n* = 100	*P value*
Age (years)	48.75 ± 12.29	47.81 ± 11.56	0.250	44.82 ± 12.50	50.51 ± 11.29	< 0.001
SBP(mmHg)	124.46 ± 16.15	130.18 ± 16.25	< 0.001	114.72 ± 16.72	129.36 ± 20.46	< 0.001
DBP (mmHg)	77.35 ± 11.07	82.42 ± 11.99	< 0.001	70.85 ± 10.03	77.79 ± 11.37	< 0.001
BMI (kg/m^2^)	24.37 ± 2.72	27.33 ± 3.03	< 0.001	22.25 ± 2.65	26.5 ± 3.57	< 0.001
ALT (U/L)	19 (15,27)	26 (19,39)	< 0.001	14 (10,18)	17 (12.25,24)	< 0.001
AST (U/L)	20 (17,24)	22 (18,27)	< 0.001	18 (15,22)	19 (17,23)	< 0.001
CREA (μmol/L)	71.83 ± 11.43	74.8 ± 11.37	< 0.001	51.76 ± 7.11	54.98 ± 7.28	< 0.001
UA (μmol/L)	338.95 ± 76.42	369.58 ± 79.33	< 0.001	246.73 ± 53.29	282.47 ± 64.58	< 0.001
TC (mmol/L)	4.59 ± 0.85	4.9 ± 1.04	< 0.001	4.53 ± 0.9	5.02 ± 0.87	< 0.001
TG (mmol/L)	1.28 (0.98,1.78)	1.86 (1.29,2.89)	< 0.001	0.93 (0.70,1.22)	1.40 (1.01,2.04)	< 0.001
HDL(mmol/L)	1.25 (1.10,1.46)	1.18 (1.03,1.37)	< 0.001	1.51 (1.32,1.76)	1.35 (1.18,1.62)	0.001
LDL(mmol/L)	2.97 ± 0.69	3.17 ± 0.72	< 0.001	2.81 ± 0.7	3.25 ± 0.71	< 0.001
FPG(mmol/L)	5.22 (4.87,5.63)	5.37 (5.00,5.87)	< 0.001	5.01 (4.76,5.30)	5.22 (4.94,5.63)	0.001
TyG	8.68 ± 0.57	9.1 ± 0.71	< 0.001	8.23 ± 0.43	8.73 ± 0.61	< 0.001
TyG-BMI	211.96 ± 30.82	249.26 ± 38.58	< 0.001	183.57 ± 26.85	231.71 ± 38.59	< 0.001

### Multivariate logistic regression analysis of factors influencing NAFLD

Collinearity diagnostics on the factors were performed in the study, and identified severe collinearity between BMI, TyG, and TyG-BMI (variance inflation factor [VIF] > 10). After removing BMI, the VIF values for the remaining factors were all less than 10 (Additional file Table S1). Because the VIF for both TyG and TyG-BMI was less than 10, even though there was similarity between the two and the latter includes the multiplication of BMI, both could still be used in analysis. The findings indicated that TyG-BMI (OR = 1.039, 95% CI 1.031–1.046) emerged as the sole independent risk factor for NAFLD (Table 2).

**Table 2:** Multivariate logistic regression analysis of factors influencing NAFLD

Influence factors	β	SE	Wald	OR	95% CI	*P* value
Sex	0.013	0.240	0.003	1.013	0.633–1.623	0.956
SBP	0.012	0.007	3.395	1.012	0.999–1.026	0.065
DBP	–0.005	0.010	0.216	0.995	0.977–1.015	0.642
ALT	0.016	0.008	3.819	1.017	1–1.033	0.051
AST	–0.014	0.015	0.798	0.987	0.958–1.016	0.372
CREA	–0.006	0.007	0.640	0.994	0.98–1.008	0.424
UA	0.002	0.001	2.083	1.002	0.999–1.004	0.149
TC	–0.181	0.292	0.383	0.835	0.471–1.479	0.536
TG	0.124	0.164	0.571	1.132	0.821–1.561	0.450
HDL	0.062	0.381	0.027	1.064	0.504–2.244	0.871
LDL	0.417	0.333	1.574	1.518	0.791–2.913	0.210
FPG	–0.073	0.079	0.873	0.929	0.797–1.084	0.350
TyG	–0.480	0.364	1.732	0.619	0.303–1.264	0.188
TyG–BMI	0.038	0.004	107.916	1.039	1.031–1.046	< 0.001

### The TyG index and TyG-BMI as predictive indicators for NAFLD

**Fig. 1:**
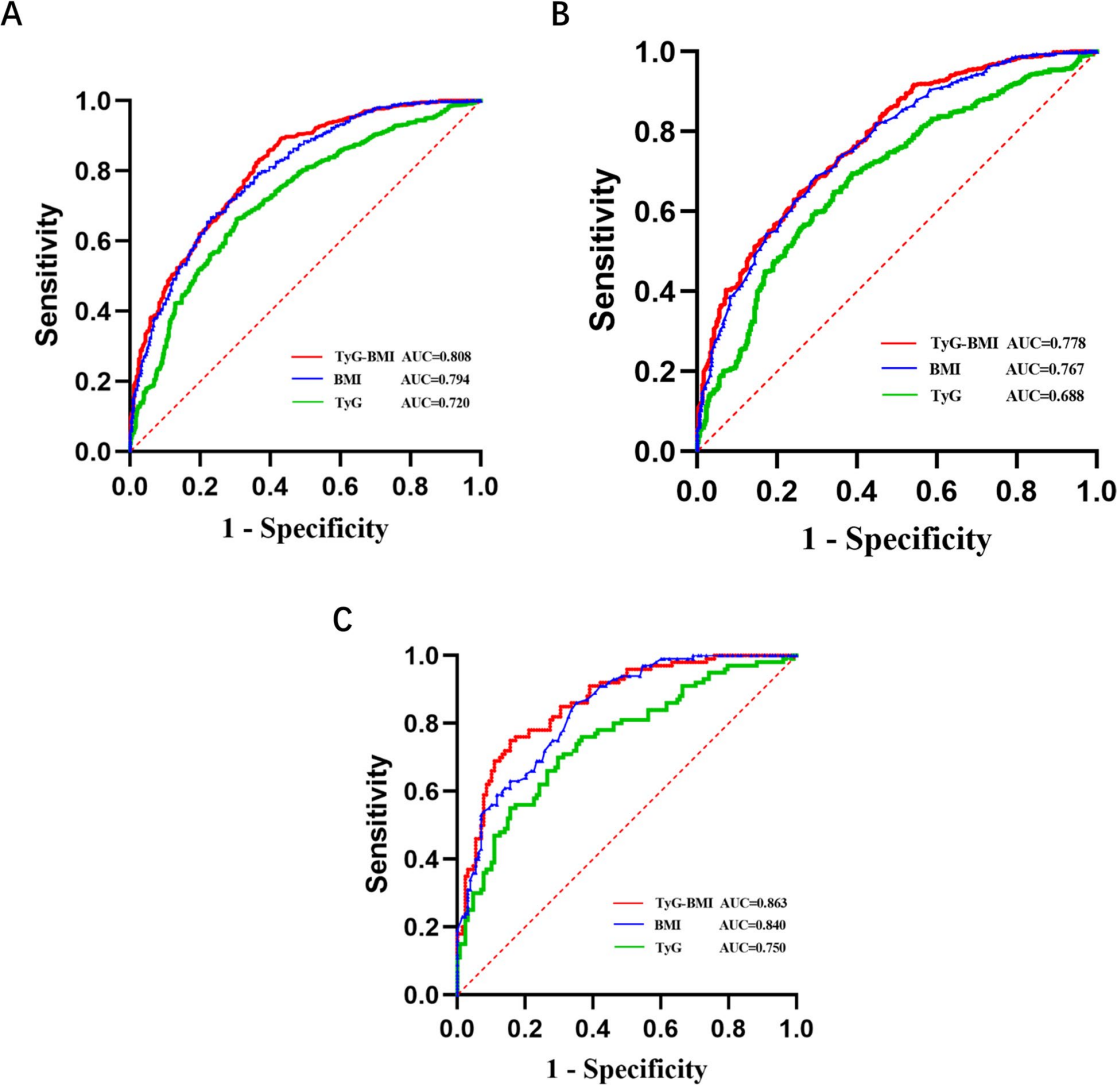
ROC curve for NAFLD-related indicators in all subjects (**A**), males (**B**) and females (**C**)

ROC curve analysis was performed with NAFLD as the state variable and baseline TyG, TyG-BMI, and BMI as test variables. The AUCs of the three indicators were 0.720, 0.808, and 0.794, indicating that all three indicators had a predictive effect on NAFLD, with TyG-BMI having the highest predictive accuracy (Fig. 1). The AUC comparisons using MedCalc observed that TyG-BMI had a slightly higher AUC than BMI (0.014 difference, Z-statistic 2.055, *P* = 0.040). The optimal cutoff for TyG was > 8.73, achieving 66.21% sensitivity and 69.59% specificity, while for TyG-BMI, a cutoff of > 213.09 yielded 83.31% sensitivity and 63.13% specificity. The positive predictive value for BMI (0.83) was higher compared to TyG-BMI (0.79), while the negative predictive value for BMI (0.57) was lower compared to TyG-BMI (0.69).

In terms of gender stratification, the analysis demonstrated that the TyG-BMI AUC value was 0.778 for males and 0.863 for females. Among the three indicators considered, TyG-BMI exhibited the highest predictive efficacy for NAFLD in both genders.

### Correlation analysis between CAP and TyG, TyG-BMI, and their components

**Fig. 2:**
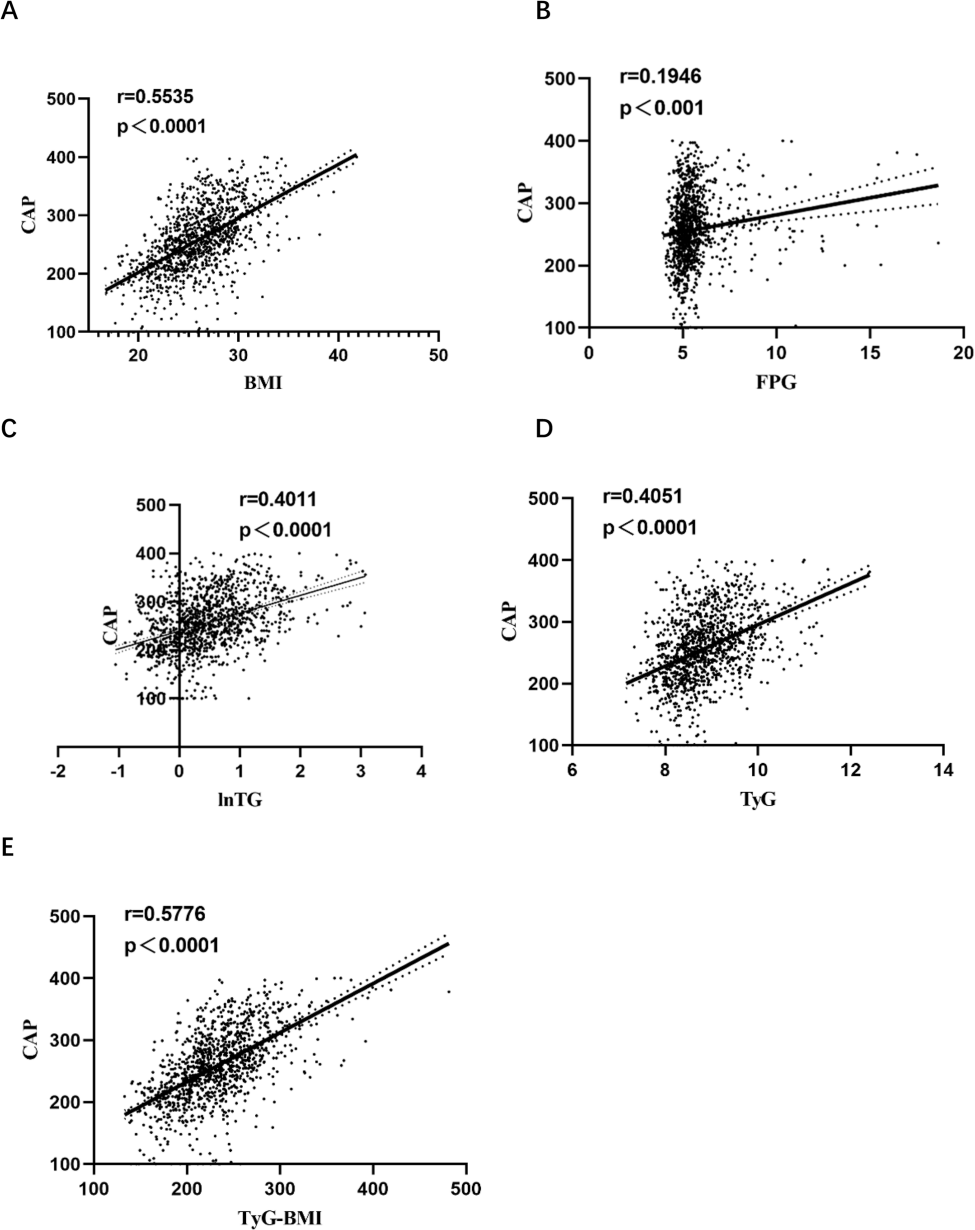
Correlation analysis between CAP values and TyG, TyG-BMI, and their components

Correlation analysis showed a positive association between CAP and BMI, FPG, lnTG, TyG, and TyG-BMI, with the highest correlation coefficient being between the CAP and TyG-BMI (Fig. 2). Due to the non-normal distribution of TG, logarithmic transformation was performed.

### Comparison of CAP between the TyG and TyG-BMI quartiles

**Fig. 3:**
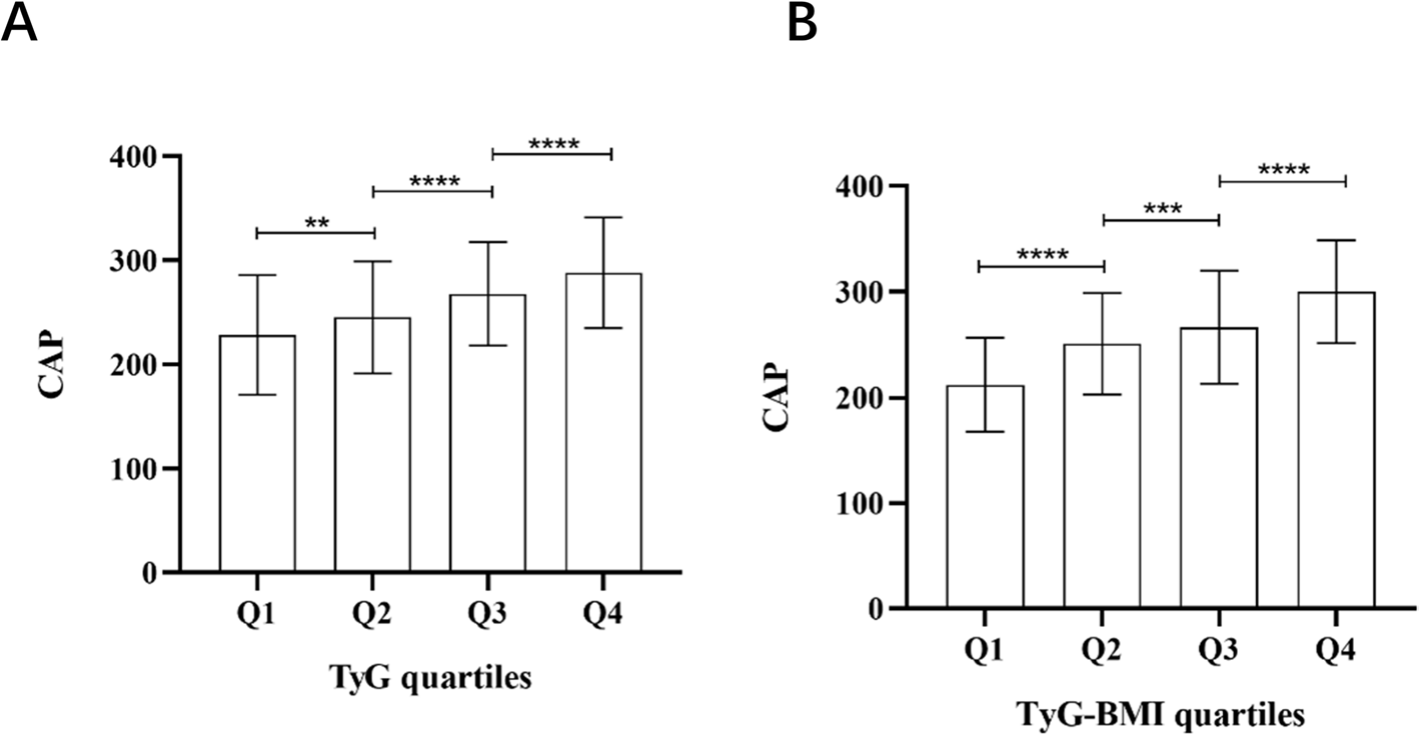
Comparison of CAP in the study population between the TyG (**A**) and TyG-BMI (**B**) quartiles. The error bars represent standard deviation (SD) Classification of TyG quartiles: Q1 (–8.36), Q2 (8.37–8.74), Q3 (8.75–9.20), Q4 (9.21–); TyG-BMI quartiles: Q1 (–200.55), Q2 (200.56–223.35), Q3 (223.36–246.62), Q4 (246.63–) **:*P* < 0.01; ***:*P* < 0.001; ****:*P* < 0.0001

The population undergoing health check-ups was divided according to the TyG and TyG-BMI quartiles. CAP values gradually increased with the increase of TyG quartiles (all *P* < 0.05) (Fig. 3A, Additional file Table S2), as well as with the increase of TyG-BMI quartiles (all *P* < 0.001) (Fig. 3B, Additional file Table S3).

### Comparison of TyG and TyG-BMI among different degrees of fatty liver

**Fig. 4:**
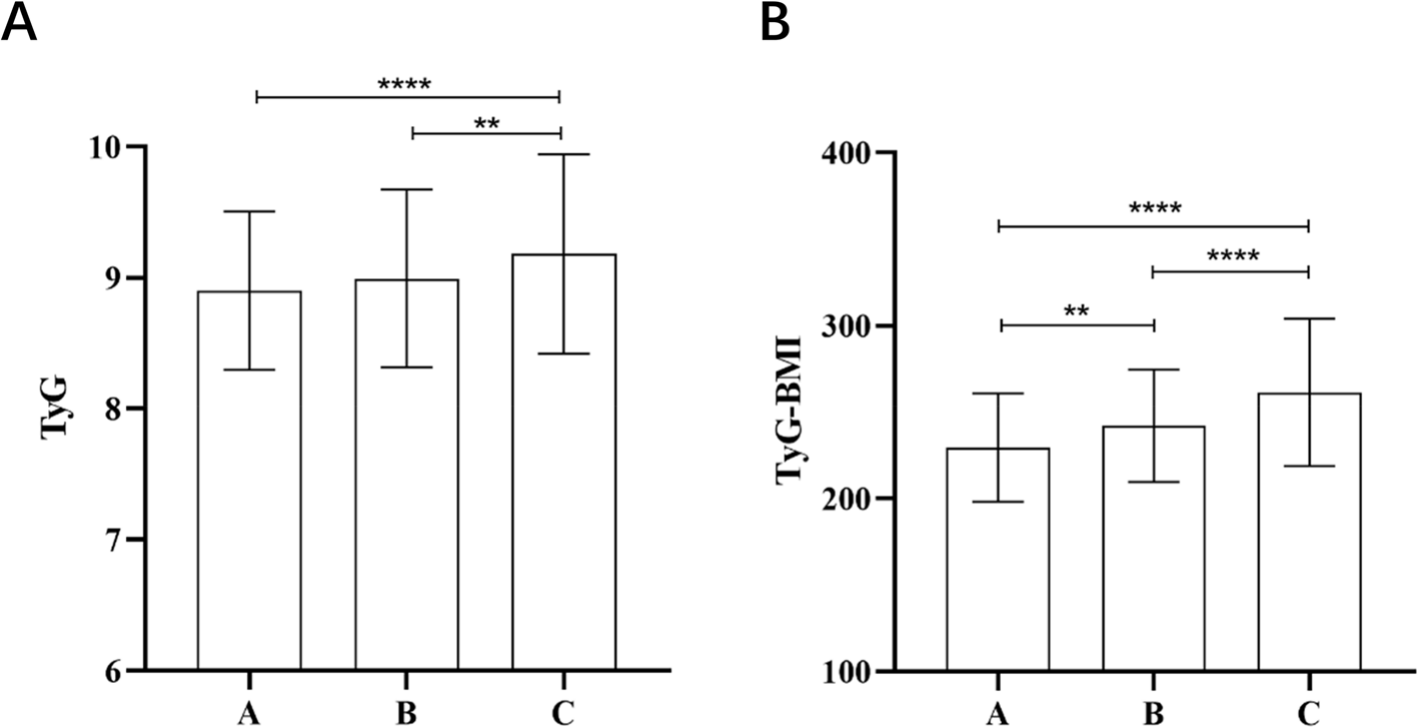
Comparison between the TyG (**A**) index and TyG-BMI (**B**) among different degrees of fatty liver. The error bars represent Standard Deviation (SD) **A**: mild fatty liver group; **B**: moderate fatty liver group; C: severe fatty liver group **:*P* < 0.01; ****:*P* < 0.0001

NAFLD patients were categorized into three groups based on the degree of fatty liver: mild (*n* = 184), moderate (*n* = 247), and severe (*n *= 306). The TyG index showed no statistically significant difference between the mild and moderate groups (*P* = 0.359) (Fig. 4A), but the TyG-BMI index showed statistically significant differences among all groups (Fig. 4B).

### Ordered multiclass logistic regression analysis of factors affecting the degree of hepatic steatosis

An ordered logistic regression analysis was conducted on the relevant factors, and the likelihood ratio test result was *P* < 0.001; in contrast, the parallel line test result was *P* = 0.430, indicating the feasibility of the analysis. The results showed that SBP (*P* = 0.007), ALT (*P* = 0.025), UA (*P* < 0.001), and TyG-BMI (*P* < 0.001) were independent factors affecting the degree of hepatic steatosis (Table 3). The higher the four indicators mentioned above, the more severe the hepatic steatosis. When TyG-BMI increased by 1 unit, the likelihood of steatosis increasing by one level was 1.034 times the original value. This likelihood was 1.031 and 1.044 times the original value for men and women, respectively. The findings revealed a positive association between the elevation of TyG-BMI and the progression of hepatic steatosis, with a greater impact observed in women compared to men.

**Table 3:** Ordered multiclass logistic regression analysis of factors affecting the degree of hepatic steatosis

Influence factors	All subjects		Male		Female	
	OR (95%CI)	*P* value	OR (95%CI)	*P* value	OR (95%CI)	*P* value
SBP (mmHg)	1.014 (1.004–1.025)	0.007	1.010 (0.999–1.022)	0.086	1.034 (1.007–1.062)	0.013
DBP (mmHg)	0.993 (0.978–1.008)	0.348	0.997 (0.981–1.014)	0.751	0.972 (0.93–1.016)	0.207
ALT(U/L)	1.013 (1.002–1.024)	0.025	1.012 (1–1.023)	0.050	1.054 (1.003–1.107)	0.037
AST(U/L)	0.986 (0.967–1.006)	0.178	0.989 (0.969–1.010)	0.306	0.929 (0.851–1.013)	0.094
CREA (μmol/L)	0.993 (0.982–1.004)	0.215	0.995 (0.984–1.007)	0.421	0.973 (0.93–1.018)	0.231
UA (μmol/L)	1.003 (1.001–1.005)	< 0.001	1.003 (1.001–1.004)	0.003	1.007 (1.001–1.012)	0.016
TC (mmol/L)	1.166 (0.785–1.733)	0.446	1.257 (0.816–1.937)	0.299	0.623 (0.157–2.471)	0.501
TG (mmol/L)	0.895 (0.77–1.041)	0.151	0.867 (0.732–1.027)	0.098	1.445 (0.425–4.906)	0.556
HDL (mmol/L)	0.681 (0.38–1.221)	0.197	0.673 (0.351–1.29)	0.233	1.351 (0.258–7.072)	0.721
LDL (mmol/L)	0.998 (0.632–1.576)	0.992	0.912 (0.553–1.504)	0.717	1.936 (0.428–8.762)	0.391
FPG (mmol/L)	0.899 (0.808–1.001)	0.053	0.905 (0.809–1.012)	0.079	0.835 (0.504–1.384)	0.485
TyG	0.835 (0.544–1.283)	0.412	0.964 (0.601–1.547)	0.881	0.337 (0.047–2.428)	0.280
TyG-BMI	1.034 (1.028–1.039)	< 0.001	1.031 (1.025–1.037)	< 0.001	1.044 (1.03–1.058)	< 0.001

## Discussion

The liver, as a significant metabolic organ, assumes critical functions in the regulation of glucose and lipid metabolism. IR and dysmetabolism of glucose and lipids are interrelated factors significantly contributing to NAFLD progression [6]. The occurrence of NAFLD can be attributed to the hepatic imbalance of triglyceride homeostasis, namely an inability to offset increased DNL or fat acid intake with fatty acid oxidation or VLDL secretion [17, 18]. Chen et al. found that residual cholesterol (RC) predominantly represented cholesterol present in the remnants of VLDL. Their findings unveiled a non-linear correlation between RC and NAFLD [19].

TyG is considered a valuable marker for IR and superior to the steady-state model IR index. TyG-related parameters, such as the TyG-BMI, have also shown predictive abilities for NAFLD [20, 21]. Nevertheless, limited research exists utilizing the TyG or TyG-BMI to predict the degree of hepatic steatosis. CAP is a non-invasive technique used on Fibroscan to identify liver steatosis; specifically, this technique assesses the presence of hepatic fat accumulation by analyzing the extent of ultrasound attenuation caused by the lipid content in the liver [22]. FibroScan has a high concordance with liver biopsy in grading liver steatosis and has consequently been endorsed as the favored non-invasive diagnostic instrument for evaluating fatty liver by major liver disease society guidelines [23, 24]. This study utilized TE to investigate whether TyG and TyG-BMI are related to the degree of liver steatosis, providing new insights into the health management of NAFLD.

The current investigation showed that the TyG-BMI played a significant role in predicting NAFLD. This study found that the optimal cutoff point for TyG was 8.73, which aligns with the results of Guo et al. They concluded that a TyG value ≥ 8.70 can effectively screen for NAFLD in the Chinese population [25]. Recent genetic and biochemical research has provided evidence supporting the significant involvement of adipose tissue in the development of IR. One potential mechanism by which adipose tissue contributes to IR is by releasing lipids and various circulating factors that propagate insulin resistance in other organs [26]. This study demonstrated that TyG-BMI is more accurate in predicting NAFLD than TyG, with an AUC of 0.808. Li et al. [27] and Kim et al. [28] discovered that the TyG-BMI had a more prominent predictive capacity for NAFLD in individuals with obesity than in those without. The study conducted by Lim et al. provided further evidence that TyG-BMI outperforms TyG or other TyG-related parameters in predicting insulin resistance [29].

This study showed a positive link between TyG, TyG-BMI, and their components with CAP. This finding aligned with the results of Khamseh et al. [13]. Furthermore, this study found that TyG-BMI was more statistically significant in evaluating the degree of liver fat deposition than TyG. Moreover, TyG-BMI was identified as the primary independent factor influencing the severity of liver fat deposition, and can be used as an effective biomarker for evaluating the severity of NAFLD. The likelihood of an increase in fatty liver by one level for every unit increase in TyG-BMI was 1.034 times higher in men than in women. However, the likelihood of an increase in women was even higher, at 1.044 times. The impact of gender on the association between TyG-BMI and the level of hepatic steatosis is not yet comprehensible. Nevertheless, this relationship could potentially be ascribed to the influence of gender on IR [30].

Being overweight and obese are major drivers of metabolic diseases and NAFLD [31]. Lipids initially accumulate in hepatocytes, leading to simple steatosis [32]. Moreover, the adipose accumulation in the liver is linked to obesity [27]. Oral et al. [33] investigated the correlation between uric acid and NAFLD. To mitigate the influence of BMI disparities between the control group and the NAFLD group, individuals with a BMI exceeding 30 were excluded. However, this study did not exclude obese cases because TyG-BMI relies on multiplying TyG and BMI. Overall, TyG-BMI has more advantages than TyG in predicting the accuracy and severity of NAFLD.

### Study strengths and limitations

This study presents several distinct strengths. First, the data for this study comes from a diverse group of individuals from different age groups and genders who underwent physical examinations, making it reasonably representative. Second, this study focuses on the impact of TyG and TyG-BMI on the degree of liver steatosis, providing new insights into health management for individuals with fatty liver. Lastly, in this investigation, analyses were carried out focusing on sex to ascertain the impact of TyG-BMI on the extent of hepatic steatosis.

However, this study is constrained by certain limitations. First, the diagnosis of NAFLD relied on TE as opposed to liver biopsy. Thus, the reliability of diagnosing NAFLD may be compromised [34]. Second, based on this cross-sectional study, it is impossible to establish the causal link between TyG and liver fat accumulation in NAFLD. Furthermore, this study was a retrospective inquiry; hence, it could not provide negative test results for hepatitis B surface antigen and hepatitis C virus antibodies. Additionally, fasting insulin and HOMA-IR were not routinely measured, thus resulting in a lack of measurement data for IR. Finally, there was no information about abdominal obesity, such as waist circumference; therefore, it was impossible to compare the parameters of the TyG combined with obesity anthropometric indicators.

## Conclusions

The results of this study suggest that TyG-BMI exhibits superior predictive accuracy for NAFLD compared to TyG alone. Additionally, a rise in TyG-BMI is correlated with an elevated degree of hepatic steatosis. Therefore, TyG-BMI proves to be a reliable indicator for identifying NAFLD within the population. NAFLD patients can reduce the risk of increasing the severity of fatty liver by lowering their TyG-BMI. Consequently, incorporating TyG-BMI into health management centers can be beneficial for monitoring and managing NAFLD.

### Supplementary Information

**Additional file 1: Table S1. **Collinearity diagnostics among various factors influencing the risk of NAFLD incidence.

**Additional file 2: Table S2. **Post-hoc test for comparing CAP among different quartiles of the TyG.

**Additional file 3: Table S3. **Post-hoc test for comparing CAP among different quartiles of the TyG-BMI.

## Data Availability

The datasets are available from the corresponding author under reasonable requests.

## Abbreviations

ALT: Alanine aminotransferase
AST: Aspartate aminotransferase
AUROC: Area under the receiver operating characteristic curve
BMI: Body mass index
CAP: Controlled attenuation parameters
CREA: Creatinine
DNL: de novo lipogenesis
DBP: Diastolic blood pressure
FPG: Fasting plasma glucose
FFAs: Free fatty acids
HDL-C: High-density lipoprotein
HOMA: Homeostasis model assessment
IR: Insulin resistance
IQR: Interquartile range
LSM: Liver stiffness measurement
LDL-C: Low-density lipoprotein
NAFLD: Non-alcoholic fatty liver disease
RC: Residual cholesterol
SBP: Systolic blood pressure
TC: Total cholesterol
TE: Transient elastography
TG: Triglycerides
TyG: Triglyceride-glucose index
UA: Uric acid
VIF: Variance inflation factor
VLDL: Very low-density lipoprotein
